# Self-Reported Rationing Behavior Among US Physicians: A National Survey

**DOI:** 10.1007/s11606-016-3756-5

**Published:** 2016-07-19

**Authors:** Robert D. Sheeler, Tim Mundell, Samia A. Hurst, Susan Dorr Goold, Bjorg Thorsteinsdottir, Jon C. Tilburt, Marion Danis

**Affiliations:** 1 Department of Family Medicine, Mayo Clinic, Rochester, MN USA; 2 University of Iowa, Iowa, USA; 3 Institute for Ethics, History, and Humanities, University of Geneva, Geneva, Switzerland; 4 Division of General Internal Medicine, Center for Bioethics and Social Sciences in Medicine, University of Michigan Medical Center, Ann Arbor, MI USA; 5 Biomedical Ethics Program, Mayo Clinic, Rochester, MN USA; 6 Division of Primary Care Internal Medicine, Mayo Clinic, Rochester, MN USA; 7 Division of General Internal Medicine, Mayo Clinic, Rochester, MN USA; 8 Knowledge and Evaluation Research Unit, Mayo Clinic, Rochester, MN USA; 9 Robert D. and Patricia E. Kern Center for the Science of Health Care Delivery, Mayo Clinic, Rochester, MN USA; 10 Department of Bioethics, National Institutes of Health, Bethesda, MD USA

**Keywords:** cost, physician attitudes, physician behavior, rationing

## Abstract

**Background:**

Rationing is a controversial topic among US physicians. Understanding their attitudes and behaviors around rationing may be essential to a more open and sensible professional discourse on this important but controversial topic.

**Objective:**

To describe rationing behavior and associated factors among US physicians.

**Design:**

Survey mailed to US physicians in 2012 to evaluate self-reported rationing behavior and variables related to this behavior.

**Setting:**

US physicians across a full spectrum of practice settings.

**Participants:**

A total of 2541 respondents, representing 65.6 % of the original mailing list of 3872 US addresses.

**Interventions:**

The study was a cross-sectional analysis of physician attitudes and self-reported behaviors, with neutral language representations of the behaviors as well as an embedded experiment to test the influence of the word “ration” on perceived responsibility.

**Main Outcome Measures:**

Overall percentage of respondents reporting rationing behavior in various contexts and assessment of attitudes toward rationing.

**Key Results:**

In total, 1348 respondents (53.1 %) reported having personally refrained within the past 6 months from using specific clinical services that would have provided the best patient care, because of health system cost. Prescription drugs (*n* = 1073 [48.3 %]) and magnetic resonance imaging (*n* = 922 [44.5 %]) were most frequently rationed. Surgical and procedural specialists were less likely to report rationing behavior (adjusted odds ratio [OR] [95 % CI], 0.8 [0.9–0.9] and 0.5 [0.4–0.6], respectively) compared to primary care. Compared with small or solo practices, those in medical school settings reported less rationing (adjusted OR [95 % CI], 0.4 [0.2–0.7]). Physicians who self-identified as very or somewhat liberal were significantly less likely to report rationing (adjusted OR [95 % CI], 0.7 [0.6–0.9]) than those self-reporting being very or somewhat conservative. A more positive opinion about rationing tended to align with greater odds of rationing.

**Conclusions:**

More than one-half of respondents engaged in behavior consistent with rationing. Practicing physicians in specific subgroups were more likely to report rationing behavior.

**Electronic supplementary material:**

The online version of this article (doi:10.1007/s11606-016-3756-5) contains supplementary material, which is available to authorized users.

## INTRODUCTION

Research has shown that physicians believe that they bear some responsibility for controlling health care costs, but tend to think that greater responsibility should be shouldered by others.^1^ Physicians have also more typically expressed the belief that they have a greater obligation to do whatever is needed for their patients than to be the primary agents for withholding health care resources for the sake of society.^1^^,^ 2

Studies have tended to focus more on physician attitudes regarding allocation of costly resources than on physician behavior. While attitudes might be a primary driver of behavior in clinical practice, this behavior may not be exclusively a function of a physician’s stated beliefs, particularly because these beliefs pertain to the sensitive topic of rationing. Little is known about self-reported rationing behavior among US physicians.

The present paper describes self-reported behavior of a random sample of US physicians consistent with a behavioral definition of rationing, and analyzes its association with demographic and practice factors. We also report an experiment designed to test for empirical evidence of social desirability bias surrounding the term “ration.” Finally, we compare self-reported rationing behavior to self-reported attitudes toward the appropriateness of rationing.

## METHODS

This study was approved by the Mayo Clinic Institutional Review Board and the Office of Human Subjects Research Protections at the National Institutes of Health Clinical Center. Its methods have been described in detail elsewhere.^1^ Briefly, we mailed a paper survey to 3872 physicians in the United States, whose names were randomly selected from the American Medical Association (AMA) Masterfile database in summer 2012. The survey, entitled “Physicians, Health Care Costs, and Society” (Online Appendix A), addressed topics related to these factors and was administered through US mail in three waves, including a cash incentive in the first mailing.

Survey measures designed to capture self-reported rationing behavior, adapted from a previous survey^3^ asking respondents to rate the frequency (never, less than monthly, monthly, weekly, daily, or not applicable) with which they “personally refrained, because of cost to the health care system, from using” 10 listed interventions—laboratory tests, routine radiography, magnetic resonance imaging (MRI), screening tests, referral to a specialist, referral to an intensive care unit, prescription drugs, referral for surgery, referral for dialysis, and hospital admission—even when that intervention would have been the best intervention for the patient. This item was intentionally worded to convey the concept of clinician-mediated rationing without using the term “rationing,” in order to minimize any potential social desirability influence on responses.

**Figure 1:**
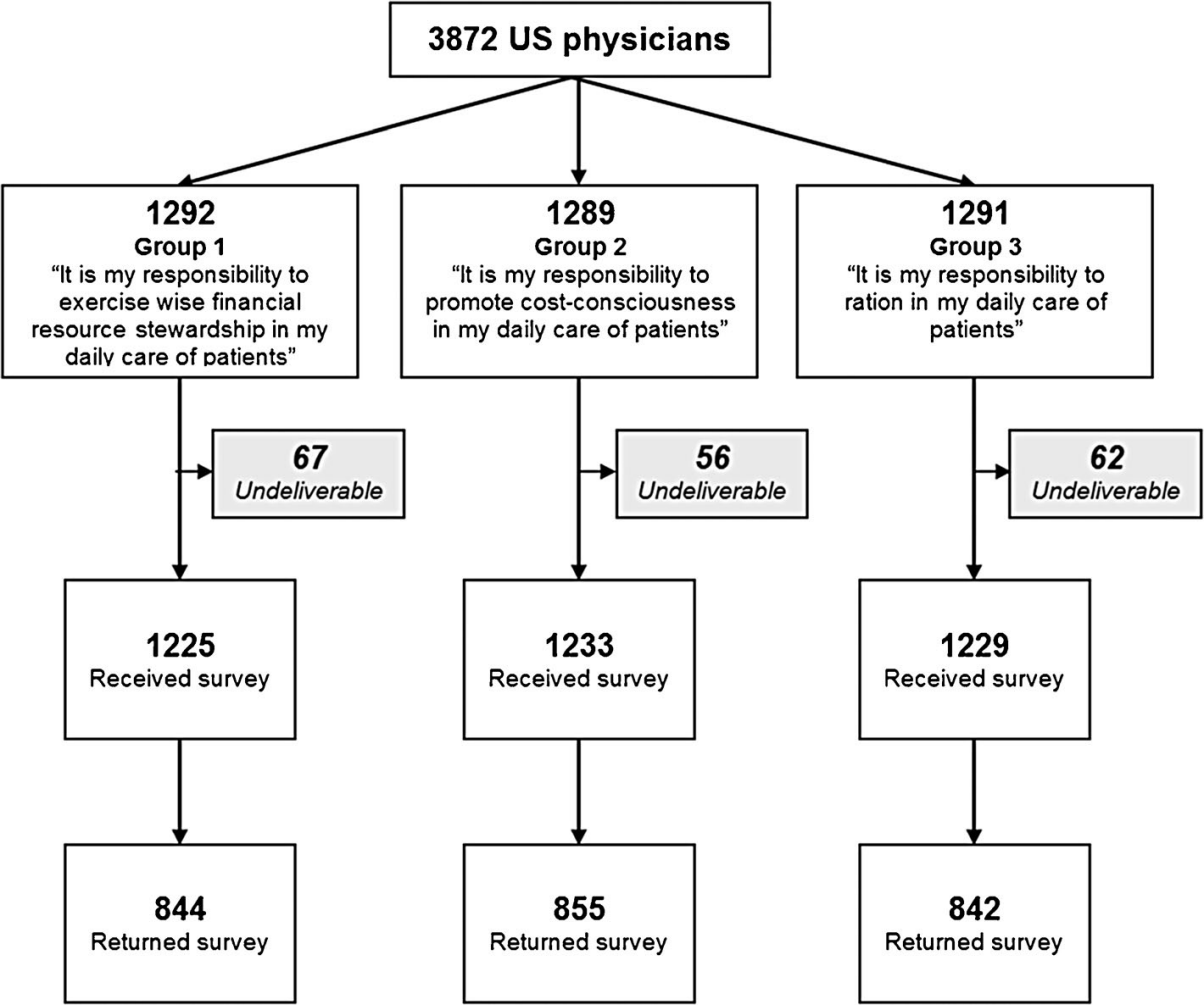
Randomization scheme for wording experiment.

To empirically test for social desirability around the term “ration,” we embedded a randomized experiment of three versions of a question item in another section of the survey about physician responsibility for containing costs. We randomly assigned each of the 3897 participants to receive an otherwise identical survey containing one of these three wording versions in one item (Fig. 1): “It is my responsibility to exercise wise financial resource stewardship in my daily care of patients”; “It is my responsibility to promote cost consciousness in my daily care of patients”; “It is my responsibility to ration in my daily care of patients.” All versions had the same response options (Strongly agree, Moderately agree, Moderately disagree, Strongly disagree) and appeared in the same place in the order of items within the survey.

### Independent Measures

Independent variables included physician demographic characteristics (i.e., age, sex, race/ethnicity, region of practice, specialty category, political self-characterization, practice setting, and compensation structure). Physician attitudes were appraised through three attitudinal items related to the appropriateness of physician roles in resource allocation (“I should sometimes deny beneficial but costly services to certain patients because resources should go to other patients that need them more”; “Cost to society is important in my decisions to use or not to use an intervention”; and “Physicians should adhere to clinical guidelines that discourage the use of interventions that have a small proven advantage over standard interventions but cost much more”), as reported previously by Hurst et al.^3^ The primary dependent variable was responding “yes” to any of the several self-reported rationing behaviors listed above.

### Data Management and Analysis

Survey responses were double-entered and imported into SAS version 9.2 (SAS Institute Inc., Cary, NC, USA). We used bivariate tests of association (Pearson *χ* ^2^ test), as well as multivariate logistic regression, to examine the association between each independent variable and self-reported rationing behavior and to analyze differences in wording that may suggest social desirability bias. We subsequently grouped specialties into the categories of primary care, surgery, procedural specialty, nonprocedural specialty, and nonclinical specialty/other. To capture self-reported rationing behavior in one variable, we subsequently dichotomized responses into one of two groups: “never” for those who reported never refraining from using any of the 10 interventions because of concern about costs (including those who selected “not applicable” or who skipped the question), and “ever” for those who reported refraining from using at least one of the interventions at any frequency (less than monthly, monthly, weekly, or daily). For adjusted associations between physician demographic characteristics and our dependent variable of interest, we created a multivariate logistic regression model containing all listed physician and practice characteristics (Model 1). Then, in a separate model, we added the three attitudinal variables together (Model 2) in which physician and practice characteristics were included as adjusting covariates. All analyses were performed using SAS version 9. Values of *p* < 0.05 were considered statistically significant.

## RESULTS

Among the 2541 survey respondents (response rate, 65.6 %), the mean age was 51.0 years, with men constituting 69.9 %, and self-reported race including 77.6 % white, 14.7 % Asian American, 3.2 % black or African American, and 4.6 % “other” (23 not reported). Respondents and non-respondents were similar with respect to sex, specialty, practice setting, and region. The mean age was slightly higher among respondents than non-respondents (51 vs. 50 years; Online Appendix B). A majority (64.5 %) practiced in group or health maintenance organization settings, followed by 19.1 % in small or solo practices, 13.2 % working for city, state, or federal governments, and 2.3 % in medical school settings. Practice types by compensation model were billing only (40.9 %), salary plus bonus (34.8 %), salary only (18.3 %), and other compensation models (6.0 %). Our physician sampling strategy did not weight for payer mix. Characteristics are summarized in Table 1.

**Table 1:** Characteristics and Attitudes Toward Rationing Among 2541 US Physician Respondents to Survey

Characteristics and attitudes	Physicians*
Age, mean (SD), years	51.0 (8.5)
Male sex	1775 (69.9)
Race/ethnicity	
White	1953 (77.6)
Asian-American	369 (14.7)
Other	116 (4.6)
Black or African-American	80 (3.2)
Not reported	23
US region	
South	829 (32.6)
Midwest	594 (23.4)
East	548 (21.6)
West	570 (22.4)
Primary specialty	
Primary care	1026 (40.4)
Surgical care	568 (22.4)
Procedural	484 (19.0)
Non-procedural	398 (15.7)
Non-clinical	44 (1.7)
Other	21 (0.8)
Practice setting type	
Group/HMO	1640 (64.5)
Small/solo	486 (19.1)
City/state/federal government	335 (13.2)
Medical school	58 (2.3)
Other	22 (0.9)
Practice compensation type	
Billing only	1027 (40.9)
Salary plus bonus	874 (34.8)
Salary only	459 (18.3)
Other	150 (6.0)
Not reported	31
Political self-characterization	
Very conservative	253 (10.2)
Somewhat conservative	708 (28.5)
Independent/moderate	720 (29.0)
Somewhat liberal/progressive	491 (19.8)
Very liberal/progressive	245 (9.9)
Other	65 (2.6)
Not reported	59
*“I should sometimes deny beneficial but costly services to certain patients because resources should go to other patients that need them more”*	
Strongly disagree	1302 (53.9)
Moderately disagree	747 (30.9)
Moderately agree	304 (12.6)
Strongly agree	61 (2.5)
Not reported	127
*“Cost to society is important in my decisions to use or not use an intervention”*	
Strongly disagree	340 (14.0)
Moderately disagree	783 (32.3)
Moderately agree	1035 (42.7)
Strongly agree	267 (11.0)
Not reported	116
*“Physicians should adhere to clinical guidelines that discourage the use of interventions that have a small proven advantage over standard interventions but cost much more”*	
Strongly disagree	101 (4.2)
Moderately disagree	400 (16.5)
Moderately agree	1117 (46.2)
Strongly agree	802 (33.1)
Not reported	121

*HMO* health maintenance organization

*Values represent the number (percentage) of physicians unless otherwise specified

For the wording experiment, response rates did not differ significantly by experimental arm. Overall, 1299 received the “wise financial stewardship” version, with 848 responding (RR = 65 %); 1298 received the “cost-conscious” version, with 861 responding (RR = 66 %); and 1300 received the “rationing” version, with 847 responding (RR = 65 %; *p* = 0.81). Respondents in all three arms of the wording experiment were similar with respect to sex, age, region, specialty, practice setting, compensation model, and self-reported political stance. (For reader convenience, a complete comparison of characteristics across groups receiving different versions of the wording is presented in Online Appendix C).

With regard to political characterization, 38.7 % of respondents considered themselves “very conservative” (10.2 %) or “somewhat conservative” (28.5 %) vs. 29.7 % as “somewhat liberal/progressive” (19.8 %) or “very liberal/progressive” (9.9 %); 29.0 % considered themselves “independent/moderate.” Table 1 summarizes the distribution of responses for attitudinal statements that solicited agreement or disagreement with rationing, as previously reported.^1^

### Self-Reported Rationing Behavior

Overall, 53.1 % of physician respondents reported that, because of the cost to the health care system, they had personally refrained from using at least one of the listed specific areas of clinical services within the prior 6 months (Table 2). The clinical services rationed most frequently were prescription drugs (48.3 %) and MRI (44.5 %). Other services were rationed considerably less often (among physicians for whom the service was available), including referral to an intensive care unit (10.9 %), referral for surgery (20.2 %), and hospital admission (18.8 %). The frequency of rationing varied considerably, but most physicians reported performing rationing less than monthly. About one-third of physicians reported rationing prescription drugs and one-fourth rationing MRIs at least monthly. In every clinical category, a small percentage of respondents (usually < %5) reported daily rationing behavior, with prescription drugs the most likely to be rationed (13.5 %).

**Table 2:** Self-Reported Rationing Behavior in the Past 6 Months Among 2541 US Physicians in Survey

Intervention	Physicians, *N* (%)					
	Not applicable or missing	Never	Less than monthly	Monthly	Weekly	Daily
Laboratory tests	279	1435 (63.4)	359 (15.9)	202 (8.9)	175 (7.7)	91 (4.0)
Routine X-ray	406	1407 (65.9)	368 (17.2)	145 (6.8)	154 (7.2)	61 (2.9)
MRI	468	1151 (55.5)	415 (20.0)	277 (13.4)	163 (7.9)	67 (3.2)
Screening test	445	1343 (64.1)	351 (16.7)	187 (8.9)	141 (6.7)	74 (3.5)
Referral to a specialist	350	1512 (69.0)	324 (14.8)	182 (8.3)	118 (5.4)	55 (2.5)
Referral to an ICU	803	1549 (89.1)	116 (6.7)	39 (2.2)	16 (0.9)	18 (1.0)
Prescription drugs	319	1149 (51.7)	289 (13.0)	239 (10.8)	244 (11.0)	301 (13.5)
Referral for surgery	470	1653 (79.8)	239 (11.5)	95 (4.6)	62 (3.0)	22 (1.1)
Referral for dialysis	1090	1334 (91.9)	77 (5.3)	18 (1.2)	11 (0.8)	11 (0.8)
Hospital admission	505	1653 (81.2)	230 (11.3)	78 (3.8)	48 (2.4)	27 (1.3)

*ICU* intensive care unit, *MRI* magnetic resonance imaging

*Note*: 220 physicians either skipped or marked “not applicable” for all 10 items

### Characteristics and Attitudes Associated With Rationing Behavior

Bivariate tests of associations (reported as unadjusted odds ratio [OR]) followed by multivariate logistic regression models (reported as adjusted OR) showed that age, sex, region of practice, and practice compensation type were not associated with reporting of ever rationing (Table 3). However, specialty and practice setting and political persuasion were significantly associated with ever having rationing behavior. Surgical and procedural specialists were distinctly less likely to report rationing (OR, 0.8 unadjusted and 0.8 adjusted for surgeons vs. 0.5 unadjusted and 0.4 adjusted for procedural specialists, *p* < 0.01 for all) compared to primary care (reference group) or nonprocedural specialties (very similar to primary care). When comparing with physicians who reported less rationing from a small or solo practice setting, we found the following unadjusted ORs: group or health maintenance organization setting, 0.8; city, state, or federal setting, 0.7; and medical school setting, 0.4 (*p* < 0.05 for all). After adjustment, the difference in rationing for the medical school setting remained significant (*p* < 0.01). Likewise, physicians who characterized their political stance as very or somewhat liberal or progressive were significantly less likely to report rationing behavior (OR, 0.7 unadjusted and 0.8 adjusted; *p* < .01) than those who reported their political stance as very or somewhat conservative.

**Table 3:** Frequency and Unadjusted/Adjusted Associations Between Characteristics, Attitudes, and Self-Reported Rationing Behavior Among 2541 US Physicians

Characteristic	*N*	Reporting “Ever rationing” in ≥1 of 10 interventions*			
		“Ever rationing” *n* (%)	Unadjusted OR (95 % CI)	Model 1^†^ adjusted OR (95 % CI)	Model 2^‡^ adjusted OR (95 % CI)
Age, years					
< 50 (ref)	1073	570 (53.1 %)	1.0	1.0	
≥ 50	1468	778 (53.0 %)	1.0 (0.8, 1.2)	1.0 (0.8, 1.2)	
Sex					
Male (ref)	1775	956 (53.9 %)	1.0	1.0	
Female	766	392 (51.2 %)	0.9 (0.8, 1.1)	0.8 (0.7, 1.0)	
Race/ethnicity					
White (ref)	1953	1014 (51.9 %)	1.0	1.0	
Asian American	369	216 (58.5 %)	1.3 (1.0, 1.6)^§^	1.4 (1.1, 1.7)^‖^	
African American	80	37 (46.3 %)	0.8 (0.5, 1.2)	0.9 (0.6, 1.4)	
Other	116	68 (58.6 %)	1.3 (0.9, 1.9)	1.2 (0.8, 1.8)	
US region					
West (ref)	570	294 (51.6 %)	1.0	1.0	
Midwest	594	324 (54.5 %)	1.1 (0.9, 1.4)	1.2 (0.9, 1.5)	
East	548	269 (49.1 %)	0.9 (0.7, 1.1)	0.9 (0.7, 1.2)	
South	829	461 (55.6 %)	1.2 (0.9, 1.5)	1.2 (1.0, 1.5)	
Political self-characterization					
Very/somewhat conservative (ref)	961	542 (56.4 %)	1.0	1.0	
Independent/moderate	720	382 (53.1 %)	0.9 (0.7, 1.1)	0.9 (0.7, 1.1)	
Very/somewhat liberal or progressive	736	357 (48.5 %)	0.7 (0.6, 0.9)^‖^	0.8 (0.6, 0.9)^‖^	
Other	65	39 (60.0 %)	1.2 (0.7, 1.9)	1.2 (0.7, 2.1)	
Specialty					
Primary care (ref)	1026	598 (58.3 %)	1.0	1.0	
Surgical	568	291 (51.2 %)	0.8 (0.6, 0.9)^‖^	0.7 (0.5, 0.8)^‖^	
Procedural	484	198 (40.9 %)	0.5 (0.4, 0.6)^‖^	0.4 (0.4, 0.6)^‖^	
Non-procedural	398	231 (58.0 %)	1.0 (0.8, 1.3)	1.0 (0.7, 1.2)	
Non-clinical/other	65	30 (46.2 %)	0.6 (0.4, 1.0)	0.7 (0.4, 1.2)	
Practice setting					
Small/solo (ref)	486	283 (58.2 %)	1.0	1.0	
Group/HMO	1640	867 (52.9 %)	0.8 (0.7, 1.0)^§^	0.9 (0.7, 1.1)	
City/state/federal government	335	170 (50.7 %)	0.7 (0.6, 1.0)*	0.8 (0.6, 1.1)	
Medical school	58	20 (34.5 %)	0.4 (0.2, 0.7)^‖^	0.4 (0.2, 0.7)^‖^	
Other	22	8 (36.4 %)	0.4 (0.2, 1.0)^§^	0.4 (0.2, 1.1)	
Compensation					
Billing only (ref)	1027	563 (54.8 %)	1.0	1.0	
Salary only	459	235 (51.2 %)	0.9 (0.7, 1.1)	0.9 (0.7, 1.1)	
Salary plus bonus	874	459 (52.5 %)	0.9 (0.8, 1.1)	1.0 (0.8, 1.2)	
Other	150	71 (47.3 %)	0.7 (0.5, 1.0)	0.7 (0.5, 1.0)	
**Attitude assessment**					
* “I should sometimes deny beneficial but costly services to certain patients because resources should go to other patients that need them more”*					
Strongly disagree (ref)	1302	625 (48.0 %)	1.0		1.0
Moderately disagree	747	442 (59.2 %)	1.6 (1.3, 1.9)^‖^		1.5 (1.3, 1.9)^‖^
Moderately agree	304	181 (59.5 %)	1.6 (1.2, 2.1)^‖^		1.5 (1.2, 2.0)^‖^
Strongly agree	61	29 (47.5 %)	1.0 (0.6, 1.6)		0.9 (0.5, 1.5)
*“Cost to society is important in my decisions to use or not use an intervention”*					
Strongly disagree (ref)	340	134 (39.4 %)	1.0		1.0
Moderately disagree	783	405 (51.7 %)	1.6 (1.3, 2.1)^‖^		1.5 (1.1, 2.0)^‖^
Moderately agree	1035	591 (57.1 %)	2.0 (1.6, 2.6)^‖^		1.7 (1.3, 2.3)^‖^
Strongly agree	267	157 (58.8 %)	2.2 (1.6, 3.0)^‖^		1.8 (1.2, 2.6)^‖^
*“Physicians should adhere to clinical guidelines that discourage the use of interventions that have a small proven advantage over standard interventions but cost much more”*					
Strongly disagree (ref)	101	40 (39.6 %)	1.0		1.0
Moderately disagree	400	212 (53.0 %)	1.7 (1.1, 2.7)^§^		1.7 (1.0, 2.7)^§^
Moderately agree	1117	594 (53.2 %)	1.7 (1.1, 2.6)^‖^		1.6 (1.0, 2.5)
Strongly agree	802	441 (55.0 %)	1.9 (1.2, 2.8)^‖^		1.6 (1.0, 2.6)^§^

*HMO* health maintenance organization, *OR* odds ratio, ref reference value

*Interventions about which participants were asked to rate their frequency of rationing were laboratory tests, routine radiograph, magnetic resonance imaging, screening test, referral to specialist, referral to intensive care unit, prescription drugs, referral for surgery, referral for dialysis, and hospital admission

^†^ Model 1 includes all physician and practice characteristics

^‡^ Model 2 includes the three attitude assessments adjusted for all physician and practice characteristics

^§^ *p* < 0.05

^‖^ *p* < 0.01

In general, in both unadjusted and adjusted analyses, the odds of self-reported rationing behavior among physicians who viewed the permissibility of rationing more favorably (i.e., moderately disagree, moderately agree, or strongly agree) were 1.5 to 1.6 times as high as those who strongly disagreed with the permissibility of rationing.

### Perceived Responsibility Differences Associated with Varying Social Desirability Wording

**Figure 2:**
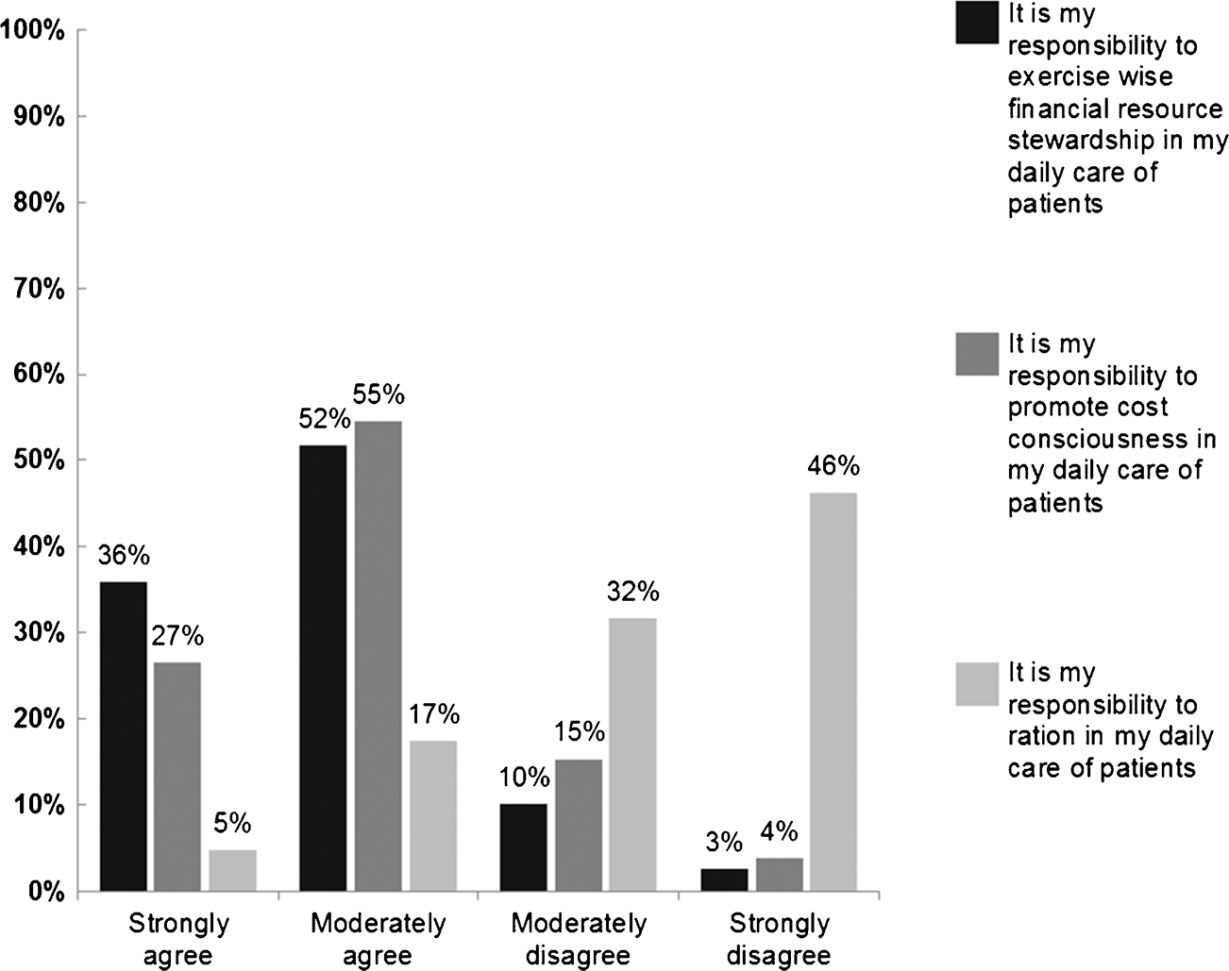
Distribution of responses to three randomized versions of wording about physicians’ responsibility for containing health care costs. *Note*: Differences between groups and overall were all statistically significant (Group 1 vs. Group 2: *p* = 0.0003; Group 1 vs. Group 3: *p* < 0.0001; Group 2 vs. Group 3: *p* < 0.0001; Group 1 vs. Group 2 vs. Group 3: *p* < 0.0001).

Respondents reported varying degrees of agreement/disagreement depending upon the question version they received. Eighty-eight percent of physicians agreed (36 % strongly and 52 % somewhat) with the “wise-stewardship” statement, and 81 % agreed (26 % strongly and 54 % somewhat) with the “cost-conscious” statement, while just 22 % agreed (5 % strongly and 17 % somewhat) with the statement containing “rationing” language (Fig. 2). The overall chi-square test comparing response distributions among the three framing versions was statistically significant (*X* ^2^ = 1025.5; *p* < 0.0001), as were all between-group differences (Group 1 vs. Group 2: *p* = 0.0003; Group 1 vs. Group 3: *p* < 0.0001; Group 2 vs. Group 3: *p* < 0.0001).

## DISCUSSION

Of the US physicians we surveyed, 53.1 % reported having rationed health care resources at least once within the past 6 months. Specialty, practice setting, and political leaning were all associated with self-reported rationing behavior. Physicians who performed procedures were less likely to report rationing behavior. The reasons for this association, however, are unclear. Specialists further downstream in care may have less ability, incentive, or need to ration. Somewhat surprisingly, physicians in small or solo practice settings were more likely to report rationing than those in group or health maintenance organizations or in a governmental context—settings that tend to be associated with more cost-consciousness. Physicians in medical school settings were least likely to report rationing. Our experimental findings lend empirical support for our approach of using a behavioral definition that avoids using the term “ration.”

We found similarities between our results and those by Hurst et al.,^3^ who assessed rationing behavior among European physicians, and on which some of the structured questions in this survey were based. In the Hurst et al. study, 56.3 % of respondents reported rationing within the past 6 months, compared with 53.1 % of our US respondents. Both the European and US physicians reported MRI scans as among the most frequently rationed services (European physicians, 40.9 %; US physicians, 44.5 %) and that intensive care unit treatment was among the least frequently rationed (European physicians, 13.7 %; US physicians, 10.9 %).^3^^–^ 5

There were significant differences in the percentage of physicians reporting rationing behavior in three general categories: (1) MRI and prescription drugs were greatest (40–50 % range); (2) laboratory tests, routine x-rays, screening tests, and specialist and surgical referrals were withheld at an intermediate rate (20–40 % range); and (3) referral to an ICU, referral for dialysis, and hospital admission were withheld the least (8–20+ % of physicians reported rationing). MRI is a high-cost item in outpatient practice and is targeted for prior authorization. Prescription drugs are often considered a separate reimbursement category and may be subject to specific active management by pharmacy benefit managers [PBMs]. On the other end of the spectrum, ICU, dialysis, and hospital admissions all represent more intensive life-or-death services, and routine withholding of these would be a more contentious matter. Whether the possible explanations raised by Hurst regarding European physicians apply to US physicians cannot be answered by these data.

Significant differences were demonstrated for all three questions indirectly asking about attitudes toward physician roles in resource allocation including rationing. One group stands out as statistically distinct: those who strongly disagreed with rationing. In the first question—“I should sometimes deny beneficial but costly services to certain patients…”—which is more individual patient-oriented, 48 % of respondents strongly disagreed, while in the latter two questions, both of which were more theoretically framed as “costs to society” and “adherence to clinical guidelines,” 39 % of respondents were in the group who strongly disagreed in each case. How we conceptualized these attitudinal variables might have influenced the associations we found.

One explanation for differences in rationing behavior among physicians in solo practice might be the *rationing by proxy* phenomenon. This term describes the situation in which physicians become rationing agents for insurance companies because of the paperwork burden and the excessive prior authorizations or out-of-pocket costs required by payers and pharmacy benefit managers. Solo practitioners who reported greater rationing behavior may have fewer resources to deal with the paperwork and other barriers; it may be easier not to make the effort in the first place when they know that their efforts will likely be in vain or will not be compensated. This response, in turn, could relate to feelings of powerlessness associated with our earlier-reported findings that, overall, physicians rated patients, health systems, and malpractice attorneys as more responsible for addressing healthcare costs than they were.^1^ Furthermore, although public discourse surrounding rationing often revolves around intensive care unit beds and expensive chemotherapy, these data on the common everyday decisions made by physicians may be as relevant or more relevant to the rationing debate.^6^^–^ 8

This study highlights the challenging nature of being a physician in the United States with regard to resource utilization. Everyday clinical decisions involve complex issues, often requiring a series of subtle judgments by an individual physician for each patient (and the patient’s family), which collectively add up to tremendous costs or cost savings. The AMA Council on Ethical and Judicial Affairs^9^ has stated, “Making cost-conscious decisions is not far removed from the professional judgments physicians already make. Physicians routinely decide whether interventions with small benefits are worthwhile,” whether ordering a laboratory stat or a routine test, choosing a brand antibiotic over a generic one, or determining how often to repeat a laboratory test. These decisions are faced several times per patient encounter within the context of larger decisions about inpatient vs. outpatient treatment, medical vs. surgical options, and resource-intensive therapies such as dialysis. The collective outcome of these decisions can mean the difference between high-cost and low-cost health care. One explanation of the discordance between opinion and behavior is the possibility of implicit, subconscious factors influencing clinical decision-making. We recognize that a variety of circumstances may cause physicians to choose a less costly service, such as the desire to reduce patient out-of-pocket expense, not all of which would fall under a narrow definition of rationing. Even with a conservative definition of what constitutes “bedside rationing,” however, we found that a significant number of US physicians reported such behavior. A broad range of behaviors that may or may not constitute bedside rationing were not captured, and these data do not provide reasons that physicians might choose to ration. At a minimum, we can say that there is a complex relationship between physicians’ attitudes toward rationing and their self-reported behaviors related to rationing. We can only speculate on the extent of complexity that future qualitative research could unpack.

To the best of our knowledge, this study is the first to focus on rationing behaviors rather than solely on attitudes regarding rationing among US physicians. The survey avoided the term “rationing” in order to assess self-reported behavior of resource allocation. Findings like these may prompt more honest and sensible professional discourse on this important but controversial topic. Although we avoided the term “rationing” in the survey, the stigma surrounding the word in the United States may have led to underreporting of actual behavior by study participants.

This study has several limitations. Estimates of specialty differences in rationing behavior should be treated with caution, because the AMA Physician Masterfile database relies on self-reported specialty data. Moreover, studying self-reported behavior does not directly address the motivations and intentions behind the behavioral choices described herein. Similarities or differences between these data and those recorded in Europe several years ago, including potential sampling differences, make it difficult to draw direct and specific comparisons. Greater granularity of information regarding the specific practice and patient population characteristics of our respondents would help to further explain the behavior. Unfortunately, such data were not available. We also acknowledge that non-response bias could be a factor in our study.^10^^,^ 11 The main contribution of this study is that it goes beyond physician-reported attitudes, to physician self-reported behavior that rationing does occur. However, with respect to self-reported rationing behavior, even if all of the non-responding physicians had reported “never” rationing, that would still mean that about 35 % of US physicians (1348/3872) reported rationing behavior. Future research could examine the disconnect between physician opinion and behavior when it comes to rationing.

We acknowledge the contentious nature of the appropriateness of physician bedside rationing. Some may not agree with the behavioral definition used in this study. US physicians report frequent rationing behavior, and there are differences in self-reported rationing based on a number of factors. Why such differences exist and the impact they have on the practice of medicine and use of resources could be explored in future research. What constitutes “justified restraint” in US health care from the perspective of the ethics of clinical medicine has been addressed by major professional societies in recent years.^9^ US physicians should be able to safely and openly discuss the ethics of rationing.

## Electronic supplementary material

Below is the link to the electronic supplementary material.

ESM 1 (PDF 272 kb)

ESM 2 (DOCX 30 kb)

ESM 3 (RTF 147 kb)

## Abbreviations

AMA: American Medical Association
MRI: Magnetic resonance imaging
OR: Odds ratio
