# Supplementary material for: Self-Reported Rationing Behavior Among US Physicians: A National Survey
Source: J Gen Intern Med. 2016 Jul 19;31(12):1444–51. doi: 10.1007/s11606-016-3756-5 (PMC5130942; doi:10.1007/s11606-016-3756-5)
Supplement: Supplementary file 1 — (PDF 272 kb) [file 11606_2016_3756_MOESM1_ESM.pdf]

Physicians, Health Care Costs, and Society

1-4 ___ ___ ___ ___

YOU & YOUR PRACTICE

Please check the appropriate box or fill in the blank as indicated.

1. How would you classify your race? (Choose ONE)

5 1 ☐ Asian or Asian-American
2 ☐ Black or African-American
3 ☐ White or Caucasian
4 ☐ Other, please specify: ____________________

2. Do you consider yourself Hispanic/Latino?

6 1 ☐ Yes
2 ☐ No

4. Which ONE of the following best describes the primary compensation for your practice?

7 1 ☐ Billing only
2 ☐ Salary only
3 ☐ Salary plus bonus
4 ☐ Other, please specify: ____________________

5. Please indicate your degree of agreement or disagreement with the following statement:
"My enjoyment of the practice of medicine is substantially lessened because of the threat of lawsuits."

8 1 ☐ Strongly disagree
2 ☐ Moderately disagree
3 ☐ Moderately agree
4 ☐ Strongly agree

6. How would you describe your average level of fatigue during the past week, including today?

9-10 0 ☐ 1 ☐ 2 ☐ 3 ☐ 4 ☐ 5 ☐ 6 ☐ 7 ☐ 8 ☐ 9 ☐ 10 ☐

(0 = No fatigue; 10 = Constant tiredness)

HEALTH CARE REFORM

Please respond to the following statements in a way that best reflects your opinions about the 2010 Patient Protection and Affordable Care Act.

7. The Affordable Care Act, if fully implemented, would turn United States health care in the right direction.

11 1☐ Strongly disagree
2☐ Moderately disagree
3☐ Moderately agree
4☐ Strongly agree

8. The Affordable Care Act, if fully implemented, would make physician reimbursement...

12 1☐ More fair
2☐ Less fair
3☐ Neither more nor less fair
4☐ Not sure

9. Should religiously affiliated institutions that object to the use of contraceptives be required to cover contraceptives in their health plans?

13 1☐ Yes
2☐ No

10. During the last 6 months, how often did you personally refrain, because of cost to the health care system, from using the following interventions when they would have been the best intervention for your patient?

		Never	Less than monthly	Monthly	Weekly	Daily	Not applicable
14	**Lab tests**	0☐	1☐	2☐	3☐	4☐	5☐
15	**Routine X-ray**	0☐	1☐	2☐	3☐	4☐	5☐
16	**MRI**	0☐	1☐	2☐	3☐	4☐	5☐
17	**Screening test**	0☐	1☐	2☐	3☐	4☐	5☐
18	**Referral to a specialist**	0☐	1☐	2☐	3☐	4☐	5☐
19	**Referral to an ICU**	0☐	1☐	2☐	3☐	4☐	5☐
20	**Prescription drugs**	0☐	1☐	2☐	3☐	4☐	5☐
21	**Referral for surgery**	0☐	1☐	2☐	3☐	4☐	5☐
22	**Referral for dialysis**	0☐	1☐	2☐	3☐	4☐	5☐
23	**Hospital admission**	0☐	1☐	2☐	3☐	4☐	5☐

PHYSICIAN RESPONSIBILITIES & SOCIETY

Please indicate your degree of agreement or disagreement with the following statements.

11. I would favor limiting coverage for expensive drugs and procedures if that would help expand access to basic health care for those currently lacking such care.

24 1 ☐ Strongly disagree
2 ☐ Moderately disagree
3 ☐ Moderately agree
4 ☐ Strongly agree

12. Every physician is professionally obligated to care for the uninsured and underinsured.

25 1 ☐ Strongly disagree
2 ☐ Moderately disagree
3 ☐ Moderately agree
4 ☐ Strongly agree

13. Addressing societal health policy issues, as important as that may be, falls outside the scope of my professional obligations as a physician.

26 1 ☐ Strongly disagree
2 ☐ Moderately disagree
3 ☐ Moderately agree
4 ☐ Strongly agree

14. Please rate the degree of responsibility (if any) each of these entities should have in reducing the cost of health care:

		No responsibility	Some responsibility	Major responsibility
27	**Government**	0 ☐	1 ☐	2 ☐
28	**Health insurance companies**	0 ☐	1 ☐	2 ☐
29	**Patients**	0 ☐	1 ☐	2 ☐
30	**Physician professional societies**	0 ☐	1 ☐	2 ☐
31	**Individual practicing physicians**	0 ☐	1 ☐	2 ☐
32	**Hospitals and health systems**	0 ☐	1 ☐	2 ☐
33	**Employers**	0 ☐	1 ☐	2 ☐
34	**Pharmaceutical and device manufacturers**	0 ☐	1 ☐	2 ☐
35	**Trial lawyers**	0 ☐	1 ☐	2 ☐

MEDICAL DECISION-MAKING

Please answer the following questions about different dimensions of medical decision-making.

15. I find the uncertainty involved in patient care disconcerting.

36 1 ☐ Strongly disagree
2 ☐ Moderately disagree
3 ☐ Moderately agree
4 ☐ Strongly agree

16. I generally order more tests when I don't know the patient well.

37 1 ☐ Strongly disagree
2 ☐ Moderately disagree
3 ☐ Moderately agree
4 ☐ Strongly agree

17. Which of the following is a major barrier to you more actively engaging patients in a process of shared decision-making? (Mark <u>ALL</u> that apply)

38-45 1 ☐ Patient confusion
1 ☐ Inability to individualize risk
1 ☐ Lack of patient interest in playing an active role
1 ☐ Lack of supportive systems (eg, computers)
1 ☐ Lack of adequate time with the patient
1 ☐ Administrative burdens
1 ☐ Financial pressure to do better paying activities (eg, procedures)
1 ☐ Other, please specify: ______________________________

18. Should promoting shared decision-making be legislated to control overall health care costs?

46 1 ☐ Yes
2 ☐ No

19. "If I tried to follow cost-conscious guidelines in my daily decision-making with individual patients..." (Mark <u>ALL</u> that apply)

47-54 1 ☐ "Patients would welcome this"
1 ☐ "It would be the right thing to do"
1 ☐ "I would not know where to start"
1 ☐ "It would be haphazard"
1 ☐ "It would likely make little difference"
1 ☐ "It could be unfair"
1 ☐ "It would likely undermine my patients' trust in me"
1 ☐ "It would help me limit unreasonable patient demands"

20. Please indicate your degree of agreement or disagreement with the following statement:
"Decision support tools that show costs would be helpful in my practice."

55 1 ☐ Strongly disagree
2 ☐ Moderately disagree
3 ☐ Moderately agree
4 ☐ Strongly agree

COST OF HEALTH CARE

A variety of practices have been proposed to control health care costs to society.

21. Please indicate your degree of enthusiasm for the following potential means of lowering health care costs (assume each is effective in lowering costs).

		Not enthusiastic	Somewhat enthusiastic	Very enthusiastic
56	Expanding access to free preventive care	0 ☐	1 ☐	2 ☐
57	Promoting head-to-head trials of competing treatments	0 ☐	1 ☐	2 ☐
58	Paying a network of practices a fixed, "bundled" price for managing all care for a defined population	0 ☐	1 ☐	2 ☐
59	Expanding electronic health records	0 ☐	1 ☐	2 ☐
60	Allowing Medicare payment cuts to doctors to take effect	0 ☐	1 ☐	2 ☐
61	Rooting out fraud and abuse	0 ☐	1 ☐	2 ☐
62	Eliminating fee-for-service payment models	0 ☐	1 ☐	2 ☐
63	Penalizing providers for avoidable readmissions	0 ☐	1 ☐	2 ☐
64	Expanding access to quality and safety data	0 ☐	1 ☐	2 ☐
65	Promoting better conversations with patients	0 ☐	1 ☐	2 ☐
66	High deductible health plans	0 ☐	1 ☐	2 ☐
67	Higher patient co-pays	0 ☐	1 ☐	2 ☐
68	Promoting continuity of care	0 ☐	1 ☐	2 ☐
69	Limiting corporate influence on physician behavior	0 ☐	1 ☐	2 ☐
70	Reducing compensation for the highest-paid specialties	0 ☐	1 ☐	2 ☐
71	Limiting access to expensive treatments with little net benefit	0 ☐	1 ☐	2 ☐
72	Promoting chronic disease care coordination	0 ☐	1 ☐	2 ☐
73	Using cost-effectiveness data to determine available treatments	0 ☐	1 ☐	2 ☐

22. Suppose a new device is proven effective at treating a serious illness compared to a placebo. If an insurance plan covers treatment for this serious illness, under which of the following circumstances, if any, would it be acceptable for the insurance plan to limit coverage for this new device? (Mark <u>ALL</u> that apply)

74-77
- 1 ☐ Never, insurance plans should cover any effective treatments for covered illnesses.
- 1 ☐ If the plan covers another treatment that is about equally effective, but costs less.
- 1 ☐ If the plan covers another treatment that is marginally less effective but costs much less.
- 1 ☐ If the plan already covers another treatment that is even more efficacious than the new device.

23. Please indicate your degree of agreement or disagreement with the following statements about health care costs:

		Strongly disagree	Moderately disagree	Moderately agree	Strongly agree
78	I am aware of the costs of the tests/treatments I recommend	1 ☐	2 ☐	3 ☐	4 ☐
79	I try not to think about the cost to the health care system when making treatment decisions	1 ☐	2 ☐	3 ☐	4 ☐
80	I should sometimes deny beneficial but costly services to certain patients because resources should go to other patients that need them more	1 ☐	2 ☐	3 ☐	4 ☐
81	I should be solely devoted to my individual patients' bests interests, even if that is expensive	1 ☐	2 ☐	3 ☐	4 ☐
82	The cost of a test or medication is only important if the patient has to pay for it out of pocket	1 ☐	2 ☐	3 ☐	4 ☐
83	Doctors are too busy to worry about costs of tests and procedures	1 ☐	2 ☐	3 ☐	4 ☐
84	Cost to society is important in my decisions to use or not to use an intervention	1 ☐	2 ☐	3 ☐	4 ☐
85	Physicians should adhere to clinical guidelines that discourage the use of interventions that have a small proven advantage over standard interventions but cost much more	1 ☐	2 ☐	3 ☐	4 ☐
86	It is my responsibility to promote cost consciousness in my daily care of patients	1 ☐	2 ☐	3 ☐	4 ☐
87	Trying to contain costs is the responsibility of every physician	1 ☐	2 ☐	3 ☐	4 ☐
88	There is currently too much emphasis on costs of tests and procedures	1 ☐	2 ☐	3 ☐	4 ☐
89	Doctors need to take a more prominent role in limiting use of unnecessary tests	1 ☐	2 ☐	3 ☐	4 ☐
90	It is unfair to ask physicians to be cost-conscious and still keep the welfare of their patients foremost in their minds	1 ☐	2 ☐	3 ☐	4 ☐

YOUR BELIEFS

In the following questions, we are interested in understanding some of your thoughts about life in general. Some items may seem odd or irrelevant, but answer each as best you can.

24. How relevant are each of the following circumstances in determining whether an action is right or wrong?

	Whether or not someone...	Not at all relevant	Not very relevant	Slightly relevant	Somewhat relevant	Very relevant	Extremely relevant
91	Suffered emotionally	0 ☐	1 ☐	2 ☐	3 ☐	4 ☐	5 ☐
92	Was treated differently than others	0 ☐	1 ☐	2 ☐	3 ☐	4 ☐	5 ☐
93	Violates standards of purity and decency	0 ☐	1 ☐	2 ☐	3 ☐	4 ☐	5 ☐
94	Is good at math	0 ☐	1 ☐	2 ☐	3 ☐	4 ☐	5 ☐
95	Cared for someone weak or vulnerable	0 ☐	1 ☐	2 ☐	3 ☐	4 ☐	5 ☐
96	Acts unfairly	0 ☐	1 ☐	2 ☐	3 ☐	4 ☐	5 ☐
97	Does something disgusting	0 ☐	1 ☐	2 ☐	3 ☐	4 ☐	5 ☐

25. Indicate your degree of agreement with the following statements based on your initial reaction.

	In life in general...	Strongly disagree	Moderately disagree	Slightly disagree	Slightly agree	Moderately agree	Strongly agree
98	Compassion for those who are suffering is the most crucial virtue	0 ☐	1 ☐	2 ☐	3 ☐	4 ☐	5 ☐
99	When the government makes laws, the number one principle should be ensuring that everyone is treated fairly	0 ☐	1 ☐	2 ☐	3 ☐	4 ☐	5 ☐
100	People should not do things that are disgusting, even if no one is harmed	0 ☐	1 ☐	2 ☐	3 ☐	4 ☐	5 ☐
101	It is better to do good than to do bad	0 ☐	1 ☐	2 ☐	3 ☐	4 ☐	5 ☐
102	One of the worst things a person could do is hurt a defenseless animal	0 ☐	1 ☐	2 ☐	3 ☐	4 ☐	5 ☐
103	Justice is the most important requirement for a society	0 ☐	1 ☐	2 ☐	3 ☐	4 ☐	5 ☐
104	Some acts are wrong on the grounds that they are unnatural	0 ☐	1 ☐	2 ☐	3 ☐	4 ☐	5 ☐
	Others' needs are more important than my own	0 ☐	1 ☐	2 ☐	3 ☐	4 ☐	5 ☐
	Government should do more to help the needy	0 ☐	1 ☐	2 ☐	3 ☐	4 ☐	5 ☐

MORE ABOUT YOU

26. Overall, how satisfied are you with practicing medicine?

105 1 ☐ Very dissatisfied
2 ☐ Somewhat dissatisfied
3 ☐ Satisfied
4 ☐ Very satisfied

27. What, if any, is your religious affiliation?

106 1 ☐ None
2 ☐ Protestant, mainline
3 ☐ Protestant, evangelical
4 ☐ Roman Catholic
5 ☐ Jewish
6 ☐ Buddhist
7 ☐ Hindu
8 ☐ Muslim
9 ☐ Other, please specify: ____________________

28. How often do you attend religious services?

107 1 ☐ Never
2 ☐ Less than once a year
3 ☐ About once or twice a year
4 ☐ Several times a year
5 ☐ About once a month
6 ☐ Two to three times a month
7 ☐ Nearly every week
8 ☐ Every week
9 ☐ Several times a week

29. Are you registered to vote?

108 1 ☐ Yes
2 ☐ No

30. How would you characterize yourself politically most of the time?

109 1 ☐ Very Conservative
2 ☐ Somewhat Conservative
3 ☐ Independent/Moderate
4 ☐ Somewhat Liberal/Progressive
5 ☐ Very Liberal/Progressive
6 ☐ Other, please specify: ____________________

Thank you for completing the survey!
Please return in the enclosed, self-addressed envelope.

MAYO CLINIC | 200 First Street SW | Rochester, MN 55905 | mayoclinic.org

©2012 Mayo Foundation for Medical Education and Research. All rights reserved. MAYO, MAYO CLINIC and the triple-shield Mayo logo are trademarks and service marks of MFMER.

MC0000-000
