# Supplementary material for: Self-Reported Rationing Behavior Among US Physicians: A National Survey
Source: J Gen Intern Med. 2016 Jul 19;31(12):1444–51. doi: 10.1007/s11606-016-3756-5 (PMC5130942; doi:10.1007/s11606-016-3756-5)
Supplement: Supplementary file 2 — (DOCX 30 kb) [file 11606_2016_3756_MOESM2_ESM.docx]

| **APPENDIX B: Non-response Analysis^a^** | | | | |
| --- | --- | --- | --- | --- |
|  | Non Responders N=1331 | Responders N=2541 | Total N=3872 | p value |
| **Age** |  |  |  | 0.0111^b^ |
| N | 1331 | 2541 | 3872 |  |
| Mean (SD) | 50.3 (8.6) | 51.0 (8.5) | 50.8 (8.5) |  |
| Median | 51.0 | 52.0 | 51.0 |  |
| Q1, Q3 | 43.0, 58.0 | 44.0, 58.0 | 44.0, 58.0 |  |
| Range | (30.0-65.0) | (30.0-65.0) | (30.0-65.0) |  |
|  |  |  |  |  |
| **Sex** |  |  |  | 0.3016^c^ |
| Female | 380 (28.5%) | 766 (30.1%) | 1146 (29.6%) |  |
| Male | 951 (71.5%) | 1775 (69.9%) | 2726 (70.4%) |  |
|  |  |  |  |  |
| **Region (including non-US regions, O)** |  |  |  | 0.2648^c^ |
| East | 274 (20.6%) | 548 (21.6%) | 822 (21.2%) |  |
| Midwest | 282 (21.2%) | 594 (23.4%) | 876 (22.6%) |  |
| South | 466 (35.0%) | 829 (32.6%) | 1295 (33.4%) |  |
| West | 309 (23.2%) | 570 (22.4%) | 879 (22.7%) |  |
|  |  |  |  |  |
| **Specialty** |  |  |  | 0.5921^c^ |
| Primary Care | 509 (38.2%) | 1026 (40.4%) | 1535 (39.6%) |  |
| Surgical Care | 291 (21.9%) | 568 (22.4%) | 859 (22.2%) |  |
| Procedural | 272 (20.4%) | 484 (19.0%) | 756 (19.5%) |  |
| Non-procedural | 225 (16.9%) | 398 (15.7%) | 623 (16.1%) |  |
| Non-clinical / Other | 34 (2.6%) | 65 (2.6%) | 99 (2.6%) |  |
|  |  |  |  |  |
| **Practice Type** |  |  |  | 0.1273^c^ |
| Small/solo | 226 (17.0%) | 486 (19.1%) | 712 (18.4%) |  |
| Group/HMO | 910 (68.4%) | 1640 (64.5%) | 2550 (65.9%) |  |
| City/state/federal government | 162 (12.2%) | 335 (13.2%) | 497 (12.8%) |  |
| Medical school | 27 (2.0%) | 58 (2.3%) | 85 (2.2%) |  |
| Other | 6 (0.5%) | 22 (0.9%) | 28 (0.7%) |  |
| ^a^ Note - excludes anyone from non-US regions  ^b^ Unequal Variance T-Test  ^c^ Chi-Square | | | | |
