# Supplementary material for: Self-Reported Rationing Behavior Among US Physicians: A National Survey
Source: J Gen Intern Med. 2016 Jul 19;31(12):1444–51. doi: 10.1007/s11606-016-3756-5 (PMC5130942; doi:10.1007/s11606-016-3756-5)
Supplement: Supplementary file 3 — (RTF 147 kb) [file 11606_2016_3756_MOESM3_ESM.rtf]

Appendix C . Characteristics of 2541 US physicians randomized to receive one of three versions of a statement pertaining to responsibilities in containing health care costs. 
	
	...exercise wise financial stwrd (N=844)	...promote cost-consciousness (N=855)	...ration in daily care (N=842)	p value	
age	 	 	 	0.58771	
    N	844	855	842	 	
    Mean (SD)	51.3 (8.7)	50.8 (8.5)	51.0 (8.3)	 	
    Median	52.0	52.0	51.0	 	
    Q1, Q3	44.0, 59.0	44.0, 58.0	45.0, 58.0	 	
    Range	(32.0-65.0)	(30.0-65.0)	(33.0-65.0)	 	
 	 	 	 	 	
Sex	 	 	 	0.30632	
    F	250 (29.6%)	274 (32.0%)	242 (28.7%)	 	
    M	594 (70.4%)	581 (68.0%)	600 (71.3%)	 	
 	 	 	 	 	
Region (including non-US regions, O)	 	 	 	0.13652	
    E	179 (21.2%)	179 (20.9%)	190 (22.6%)	 	
    MW	187 (22.2%)	215 (25.1%)	192 (22.8%)	 	
    S	291 (34.5%)	251 (29.4%)	287 (34.1%)	 	
    W	187 (22.2%)	210 (24.6%)	173 (20.5%)	 	
 	 	 	 	 	
spec1b	 	 	 	0.12542	
    1_PC	353 (41.8%)	325 (38.0%)	348 (41.3%)	 	
    2_S	197 (23.3%)	194 (22.7%)	177 (21.0%)	 	
    3_PR	151 (17.9%)	168 (19.6%)	165 (19.6%)	 	
    4_NP	128 (15.2%)	135 (15.8%)	135 (16.0%)	 	
    5_NC/O	15 (1.8%)	33 (3.9%)	17 (2.0%)	 	
 	 	 	 	 	
practice2	 	 	 	0.36862	
    1_small	170 (20.1%)	160 (18.7%)	156 (18.5%)	 	
    2_group	551 (65.3%)	541 (63.3%)	548 (65.1%)	 	
    3_govnt	95 (11.3%)	127 (14.9%)	113 (13.4%)	 	
    4_medsc	17 (2.0%)	22 (2.6%)	19 (2.3%)	 	
    5_othr	11 (1.3%)	5 (0.6%)	6 (0.7%)	 	
					
Primary compensation for your practice	 	 	 	0.83262	
    Missing	9	12	10	 	
    1 Billing only	351 (42.0%)	337 (40.0%)	339 (40.7%)	 	
    2 Salary only	142 (17.0%)	166 (19.7%)	151 (18.1%)	 	
    3 Salary + bonus	289 (34.6%)	289 (34.3%)	296 (35.6%)	 	
    4 Other	53 (6.3%)	51 (6.0%)	46 (5.5%)	 	
 	 	 	 	 	
How would you characterize yourself politically	 	 	 	0.87502	
most of the time?	 	 	 	 	
    Missing	25	14	20	 	
    1 Very conservative	75 (9.2%)	92 (10.9%)	86 (10.5%)	 	
    2 Somewhat conservative	234 (28.6%)	245 (29.1%)	229 (27.9%)	 	
    3 Independent/moderate	252 (30.8%)	232 (27.6%)	236 (28.7%)	 	
    4 Somewhat liberal/progressive	157 (19.2%)	164 (19.5%)	170 (20.7%)	 	
    5 Very liberal/progressive	83 (10.1%)	86 (10.2%)	76 (9.2%)	 	
    6 Other	18 (2.2%)	22 (2.6%)	25 (3.0%)	 	
 	 	 	 	 	
Perceived Responsibility (v1_86)	 	 	 	<0.00012	
	 	 	 	 	
    Missing	33	36	54	 	
    1 Strongly disagree	20 (2.5%)	31 (3.8%)	364 (46.2%)	 	
    2 Mod. disagree	81 (10.0%)	125 (15.3%)	249 (31.6%)	 	
    3 Mod. agree	419 (51.7%)	446 (54.5%)	137 (17.4%)	 	
    4 Strongly agree	291 (35.9%)	217 (26.5%)	38 (4.8%)	 	
(report generated on 22MAR2016)
1ANOVA F-Test    2Chi-Square	
